# A novel application of E1A in combination therapy with EGFR-TKI treatment in breast cancer

**DOI:** 10.18632/oncotarget.11737

**Published:** 2016-08-31

**Authors:** Chih-Ming Su, Ting-Yu Chang, Hui-Ping Hsu, Hui-Huang Lai, Jie-Ning Li, Yu-Jhen Lyu, Kuang-Tai Kuo, Ming-Te Huang, Jen-Liang Su, Pai-Sheng Chen

**Affiliations:** ^1^ Division of General Surgery, Department of Surgery, School of Medicine, College of Medicine, Taipei Medical University, Taipei City, Taiwan, ROC; ^2^ Division of General Surgery, Department of Surgery, Shuang Ho Hospital, Taipei Medical University, New Taipei City, Taiwan, ROC; ^3^ National Institute of Cancer Research, National Health Research Institutes, Zhunan, Miaoli Country, Taiwan, ROC; ^4^ Department of Surgery, National Cheng Kung University Hospital, Tainan, Taiwan, ROC; ^5^ Institute of Basic Medical Sciences, College of Medicine, National Cheng Kung University, Tainan, Taiwan, ROC; ^6^ Department of Medical Laboratory Science and Biotechnology, College of Medicine, National Cheng Kung University, Tainan, Taiwan, ROC; ^7^ Division of Thoracic Surgery, Department of Surgery, Shuang Ho Hospital, Taipei Medical University, New Taipei City, Taiwan, ROC; ^8^ Division of Thoracic Surgery, Department of Surgery, School of Medicine, College of Medicine, Taipei Medical University, Taipei City, Taiwan, ROC; ^9^ Center for Molecular Medicine, China Medical University Hospital, Taichung, Taiwan, ROC; ^10^ Graduate Institute of Cancer Biology, China Medical University, Taichung, Taiwan, ROC; ^11^ Department of Biotechnology, Asia University, Taichung, Taiwan, ROC

**Keywords:** combination therapy, E1A

## Abstract

Epidermal growth factor receptor (EGFR) is commonly overexpressed in breast cancer and is associated with poor clinical outcomes; however, an increasing number of patients have shown a poor effective response to EGFR tyrosine kinase inhibitors (EGFR-TKI). Here, we found that AXL expression was positively correlated with poor progression in breast cancer patients. Suppression of AXL by an anti-tumor protein, E1A, enhanced EGFR-TKI (gefitinib, erlotinib and lapatinib) sensitization, resulting in significant inhibition of tumor growth in breast cancer cells. Additionally, AXL overexpression dramatically impaired E1A-mediated EGFR-TKI sensitization. These findings show that downregulation of AXL expression by E1A contributes to sensitization to EGFR-TKI in breast cancer, suggesting that combinatorial therapy of AXL inhibitors or E1A gene therapy with EGFR-TKI may be a potential therapeutic strategy for treatment of breast cancer patients.

## INTRODUCTION

EGFR is one of four structurally related receptor tyrosine kinases (RTKs) of the ErbB family [1]. Recent studies have shown that overexpression of EGFR is commonly found in breast cancer and is correlated with poor prognosis; thus, EGFR may be a potential therapeutic target [2]. In clinical studies, patients with non-small cell lung cancer who received EGFR-TKI (gefitinib and erlotinib) treatment showed a good therapeutic response [3]. However, it was disappointing that breast cancer patients did not respond well to EGFR inhibitors alone [4, 5]. A combination of gefitinib and endocrine therapy or chemotherapeutic drugs has been used in various phase I and II clinical trials [6–8]. In addition, lapatinib in combination with chemotherapy showed an increased efficacy in advanced breast cancer but was associated with toxicity [9]. Therefore, treatment with EGFR inhibitors alone appears to be insufficient, and the activation of EGFR or downstream signaling may be through alternative oncogenic pathways causing resistance [10, 11]. A combination of EGFR-TKI with other treatments might be a useful strategy in breast cancer therapy.

Adenovirus-5 early region 1A (E1A) exerts its anti-tumor activity through various mechanisms, such as increasing drug sensitivity through the downregulation of HER2/neu [12, 13]; enhancing chemosensitivity by activating the tumor suppressor gene p53 [14]; inducing apoptosis through the activation of p38 [15]; decreasing the levels of miR-520h and the EMT marker TWIST to inhibit metastasis [16]; and increasing chemosensitization by stabilizing FOXO3a [17]. Moreover, E1A has been shown to downregulate RTKs, such as EGFR, HER2/neu or AXL [18–20], and accumulating evidence has indicated that RTKs play important roles in cancer progression. Thus, there may be a potential synergistic effect with E1A and EGFR-TKI combinational therapy by blocking the crosstalk between EGFR and other TKs in treatment of breast cancer.

AXL, a receptor tyrosine kinase is a member of the TAM (Tyro-Axl-Mer) family, that is overexpressed and activated in various human cancers [21] and is correlated with poor prognosis in patients. Inhibition of AXL decreased migration and invasion in breast cancer cells [22] and reduced cancer cell proliferation, survival, and tumor growth [23–25]. In addition, several studies reported that AXL activation was involved in resistance to EGFR-TKI [26] and chemotherapeutic drugs, such as cisplatin, doxorubicin or sulfasalazine [27–29]. EGFR activation induced transactivation of AXL, which leads to diversification of downstream signaling and contributes to RTK-targeted drug resistance [30]. Collectively, AXL may play a crucial role in resistance to EGFR inhibitors and may represent a critical therapeutic strategy for breast cancer.

In this study, we found that inhibition of AXL (E1A gene therapy or R428) increases the sensitization of breast cancer cells to EGFR-TKI. Notably, we also found that higher expression of AXL is correlated with poor overall survival, advanced tumor stage, and lymph node status of breast cancer patients. Taken together, our findings suggest that a combination of E1A gene therapy or an AXL inhibitor and EGFR-TKI will improve treatment of breast cancer. These results suggest that combinational therapeutic strategies for treatment of breast cancer patients should be developed.

## RESULTS

### AXL is a poor prognostic marker which correlates with advanced stages of breast cancer

**Figure 1:**
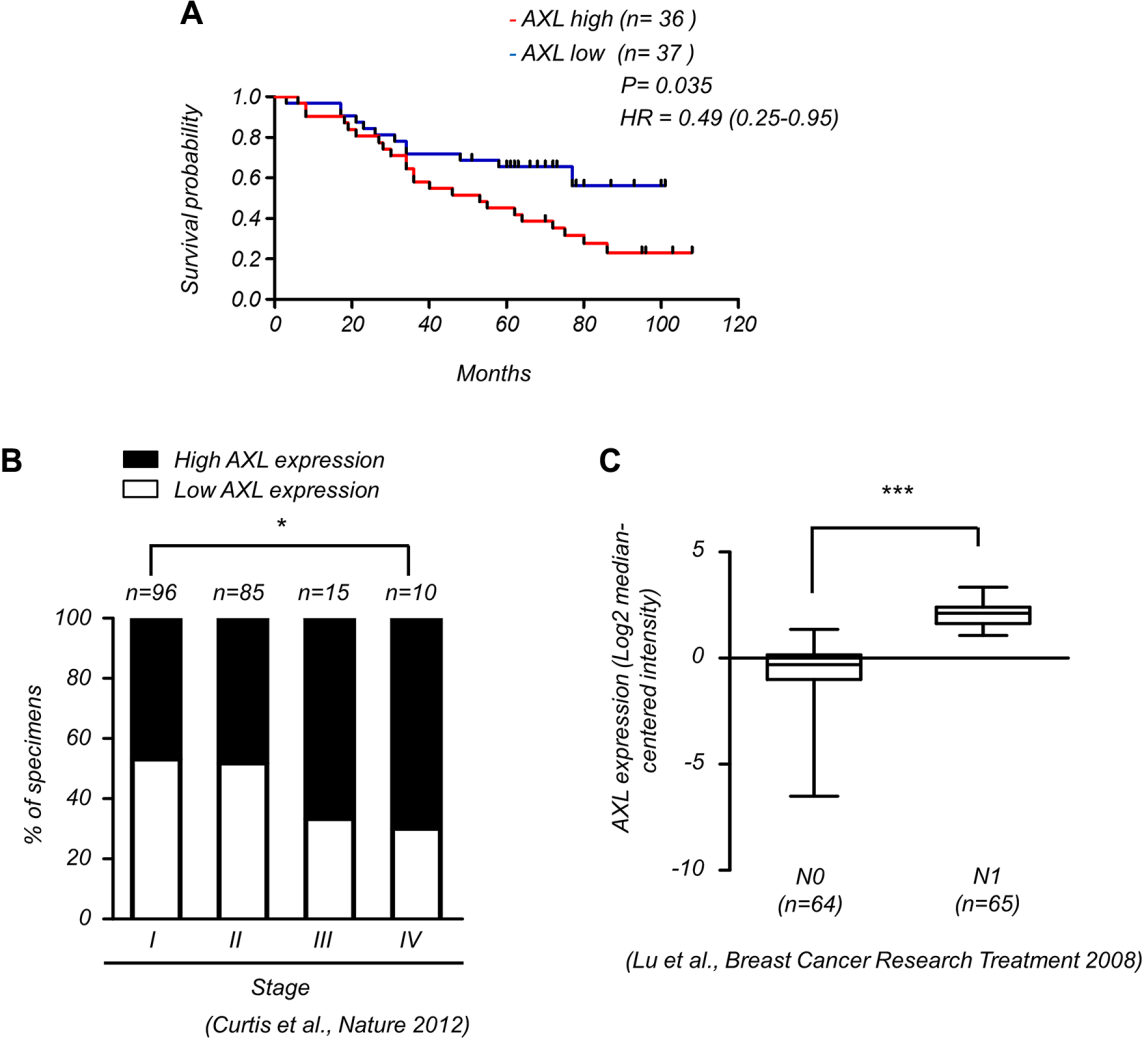
Expression of AXL correlates with malignant progression of breast cancer (**A**) Kaplan-Meier analysis of the overall survival of 73 breast cancer patients with low and high expression of AXL (*P* = 0.035, log-rank test, HR = 0.49). AXL expression in tumors was classified according to median of the individual ΔCt values of patient samples. The median of individual ΔCt values of patient samples was used as the cut-off value between high and low expression. Lower ΔCt values indicate higher expression of the gene. (**B**) AXL expression correlates with high stages of breast cancer specimens (Oncomine datasets: Curtis_Breast). The number (n) of patients for each stage is indicated at the top of each column; *P < 0.05. (**C**) AXL expression positively correlated with lymph node status in breast cancer patients (Oncomine datasets: Lu_Breast).

To elucidate the clinical significance of AXL expression in breast cancer patients, we analyzed a cohort of 73 breast cancer patients by Kaplan-Meier survival analysis and observed that high levels of *AXL* expression (*n* = 36) were significantly correlated with poor survival outcomes compared to those with low levels of *AXL* (*n* = 37) (Figure 1A). The Oncomine database also showed that *AXL* expression was positively correlated with advanced tumor stages (Figure 1B) and lymph node status (Figure 1C) of breast cancer patients, suggesting that *AXL* is crucial for tumor progression and survival outcomes in breast cancer patients.

### Suppression of AXL enhances EGFR-TKI cytotoxicity in breast cancer cells

**Figure 2:**
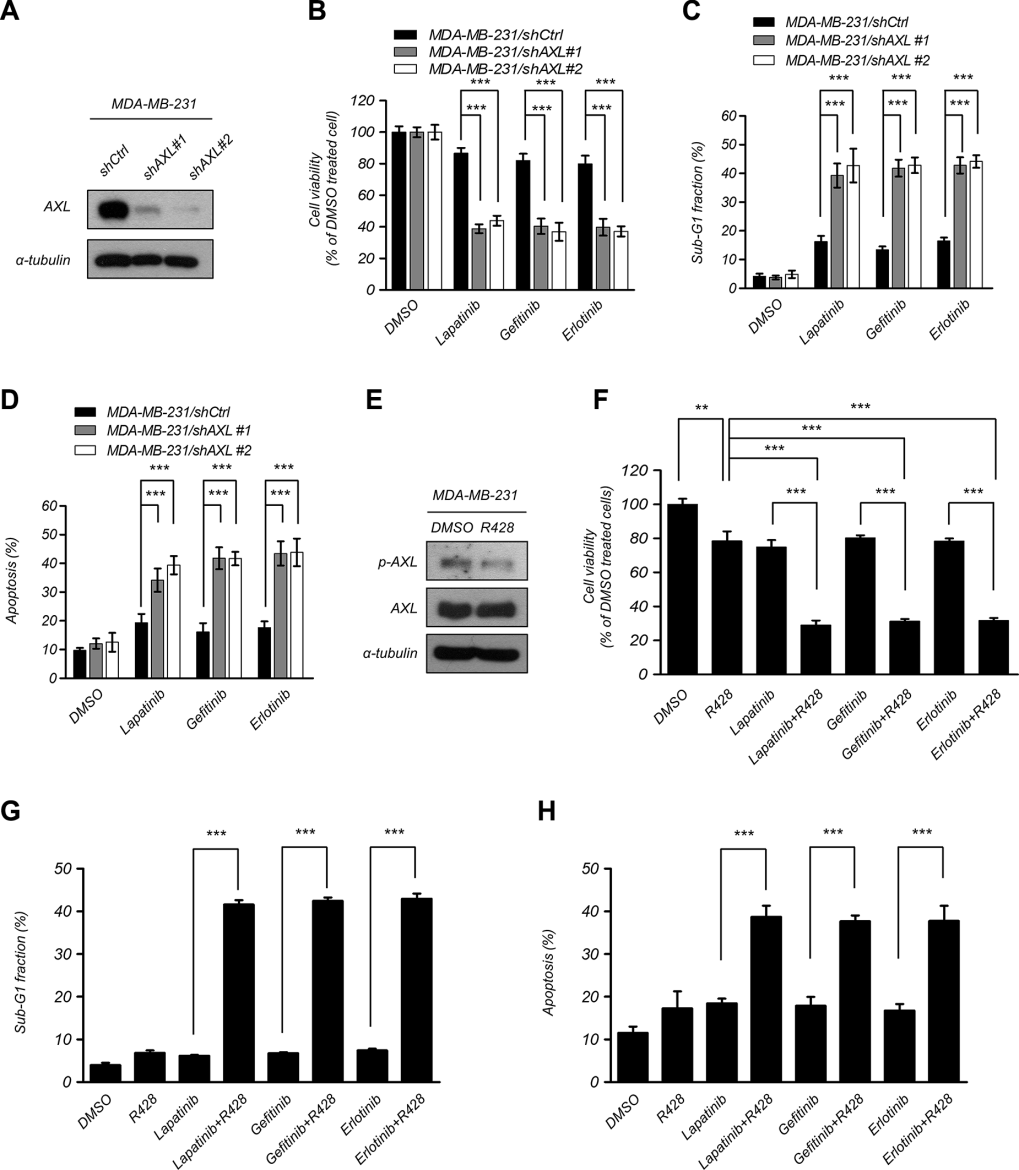
Suppression of AXL enhanced EGFR-TKI cytotoxicity in breast cancer cells (**A–C**) Knockdown of AXL expression in MDA-MB-231 cells and detection of protein expression using western blotting analysis, measurement of the cell proliferation using MTT assays and assessment of cell death in sub-G1 phase using flow cytometry analysis after treatment with EGFR-TKI (2.5 μM lapatinib, 10 μM gefitinib, and 20 μM erlotinib) for 48 h. The columns represent the mean values from 3 independent experiments. Bars indicate the means ± s.e.m. ***P < 0.001. (**D**) Cells were treated with EGFR-TKI (2.5 μM lapatinib, 10 μM gefitinib, and 20 μM erlotinib) for 48 h and were assayed by double staining of PI and Annexin V and were then analyzed using flow cytometry. Both Annexin V + /PI − (early apoptosis) and Annexin V + /PI + (late apoptosis) cells were considered as apoptotic cells. The columns represent the mean percentages of apoptotic cells Annexin V-FITC positive cells from 3 independent experiments. Bars indicate the means ± s.e.m. ***P < 0.001. (**E–G**) Treatment with an AXL inhibitor, R428 (10 nM), for 48 h in MDA-MB-231 cells. Phosphorylation and total AXL protein expression were analyzed using western blotting analysis. The cell viability was analyzed using MTT assays, and the percentage of sub-G1 cells was analyzed using flow cytometry analysis after treatment with EGFR-TKI. The columns represent the mean values from 3 independent experiments. Bars indicate the means ± s.e.m. **P < 0.01, ***P < 0.001. (**H**) MDA-MB-231 cells were treated with R428 (10 nM) and EGFR-TKI (2.5 μM lapatinib, 10 μM gefitinib, and 20 μM erlotinib) for 48 h. Cell apoptosis was assayed by double staining of PI and Annexin V and was than analyzed using flow cytometry. Both Annexin V + /PI − (early apoptosis) and Annexin V + /PI + (late apoptosis) cells were considered as apoptotic cells. Data represent the mean values of apoptotic percentages from 3 independent experiments. Bars indicate the means ± s.e.m. ***P < 0.001.

To confirm whether AXL inhibition enhances the sensitization to EGFR-TKI, we knocked down AXL by specific short hairpin RNA (shRNA) in MDA-MB-231 and HBL100 cells and determined the cell viability after treatment with EGFR-TKI (Figure 2A and Supplementary Figure S1A). Suppression of AXL significantly decreased cell viability after EGFR-TKI treatment compared with the control cells (Figure 2B and Supplementary Figure S1B). We also performed flow cytometry to analyze the percentage of sub-G1 cells after treatment with EGFR-TKI and found that depleting expression of AXL in MDA-MB-231 cells significantly increased cell death and apoptosis (Figure 2C–2D and Supplementary Figure S1C). To verify the effects of AXL inhibition in combination with EGFR-TKI, cells were treated with a selective small molecule inhibitor of AXL, R428, to suppress the activation of AXL [31]. The results showed that R428 treatment led to inactivation of AXL in MDA-MB-231 and HBL100 cells (Figure 2E and Supplementary Figure S1D). After a combination treatment of R428 and EGFR-TKI, cells were found to be more sensitive to the EGFR-TKI treatment compared with R428 or EGFR-TKI alone (Figure 2F and Supplementary Figure S1E). In addition, cell death in sub-G1 phase and cell apoptosis were enhanced in MDA-MB-231 cells that received the combinational treatment (Figure 2G–2H). These findings indicate that the suppression of AXL enhances EGFR-TKI efficacy in human breast cancer cells, suggesting that AXL plays a functional role in mediating EGFR-TKI sensitization in breast cancer cells.

### Sensitization of breast cancer cells to EGFR-TKI treatment by E1A

**Figure 3:**
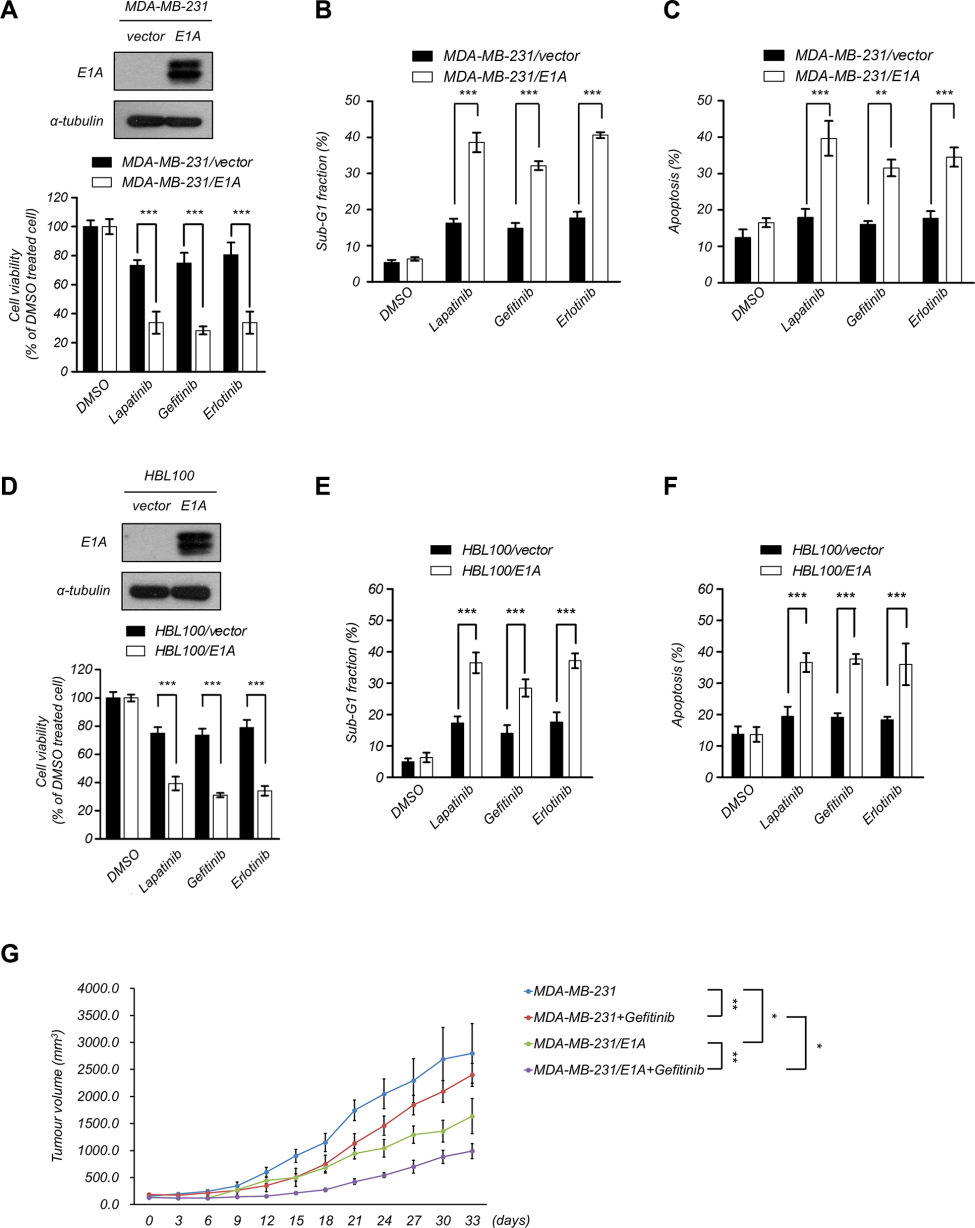
E1A sensitizes breast cancer cells to EGFR-TKI treatment (**A**) E1A enhances EGFR-TKI sensitization in MDA- MB- 231 cells. Top, the protein expression of E1A in E1A-expressing cells and vector-expressing cells was analyzed using western blotting analysis. Bottom, cell proliferation was determined by MTT assays after treatment with EGFR-TKI (2.5 μM lapatinib, 10 μM gefitinib, and 20 μM erlotinib) for 48 h. The columns show the mean values from 3 independent experiments. Bars indicate the means ± s.e.m. ***P < 0.001. (**B**) The percentage of sub-G1 cells was analyzed by flow cytometry analysis after treatment with EGFR-TKI (2.5 μM lapatinib, 10 μM gefitinib, and 20 μM erlotinib) for 48 h. The columns show the mean values from 3 independent experiments. Bars indicate the means ± s.e.m. ***P < 0.001. (**C**) MDA-MB-231 and MDA-MB-231/E1A cells were treated with EGFR-TKI (2.5 μM lapatinib, 10 μM gefitinib, and 20 μM erlotinib) for 48 h and cell apoptosis was examined by double staining of PI and Annexin V and was than analyzed using flow cytometry. Both Annexin V + /PI – (early apoptosis) and Annexin V + /PI + (late apoptosis) cells were considered as apoptotic cells. The columns show the mean values from 3 independent experiments. Bars indicate the means ± s.e.m. ***P < 0.001. (**D**) E1A enhances EGFR-TKI sensitization in HBL100 cells. Top, the protein expression of E1A and α-Tubulin was analyzed using western blotting analysis. Bottom, cell proliferation was determined using MTT assays after treatment with EGFR-TKI for 48 h. The columns show the mean values from 3 independent experiments. Bars indicate the means ± s.e.m. ***P < 0.001. (**E**) The percentage of sub-G1 cells was analyzed by flow cytometry analysis in HBL100/E1A cells compared with HBL100/vector control cells after treatment with EGFR-TKI for 48 h. The columns represent the mean values from 3 independent experiments. Bars indicate the means ± s.e.m. ***P < 0.001. (**F**) Cell apoptosis was examined by double staining of PI and Annexin V and was then analyzed using flow cytometry in HBL100/E1A cells compared with HBL100/vector control cells after treatment with EGFR-TKI for 48 h. Both Annexin V + /PI – (early apoptosis) and Annexin V + /PI + (late apoptosis) cells were considered as apoptotic cells. The columns represent the mean values from 3 independent experiments. Bars indicate the means ± s.e.m. ***P < 0.001. (**G**) MDA-MB-231/vector or MDA-MB-231/E1A cells were orthotopically injected into SCID mice. Tumor volumes measured after combination treatment with gefitinib every three days are showed (*n* = 6 per group). Results are represented as the means ± s.e.m. *P < 0.05, **P < 0.01.

Both EGFR and AXL play important role in cancer progression and are related to poor prognosis. The activation of AXL contributes to resistance to EGFR-TKI in multiple types of cancers [11, 26, 30, 32, 33]. To examine the correlation between EGFR and AXL expression in breast cancer patients, we analyzed the published clinical data using the Oncomine database (http://www.oncomine.org) and found that the expression of EGFR is positively correlated with AXL in breast cancer patients (Table 1). To investigate whether E1A would enhance the sensitization to EGFR-TKI in breast cancer cells, we examined the sensitivity to lapatinib, gefitinib or erlotinib in MDA-MB-231 and HBL100 cells with ectopic expression of E1A. We found that E1A-transfected cells were more sensitive to lapatinib, gefitinib and erlotinib than the vector control cells (Figure 3A–3C). E1A treatment in combination with EGFR-TKI in HBL100 cells also showed dramatically inhibited cell viability and increased cell death (Figure 3D–3F). Similar effects were observed in animal experiments, as after gefitinib treatment, the tumor volumes of MDA-MB-231/E1A -injected mice were significantly reduced comparing to MDA-MB-231/vector-injected mice (Figure 3G). These results suggest that E1A enhances the sensitization of breast cancer cells to EGFR-TKI both *in vitro* and *in vivo*.

**Table 1:** Association between *EGFR* and *AXL*expression in breast cancer datasets

*EGFR* vs.	Datasets	^†^Pearson correlation,*r*	^‡^*P*-value
*AXL*	Boersma (*n* = 96)	0.6426	< 0.0001
	Bonnefoi (*n* = 160)	0.4404	< 0.0001
	Bos (*n* =204)	0.1974	0.0046
	Chin (*n* =118)	0.3656	< 0.0001
	Curtis (*n* = 2136)	0.0901	< 0.0001
	Desmedt (*n* = 198)	0.1523	0.0322
	Farmer (*n* = 49)	0.3558	0.0121
	Ginestier (*n* = 55)	0.4279	0.0011
	Hatzis (*n* = 508)	0.4539	< 0.0001
	Ivshiva (*n* = 289)	0.1688	0.0040
	Kao (*n* =327)	0.1580	0.0042
	Loi (*n* =87)	0.3515	0.0008
	Lu (*n* =129)	0.2388	0.0064
	Ma (*n* =66)	0.3110	0.0110
	Miller (*n* = 116)	0.2291	0.0134
	Minn (*n* =121)	0.5903	< 0.0001
	Miyake (*n*=115)	0.2117	0.0232
	Tabchy (*n*=178)	0.6300	< 0.0001

† *r*, Pearson's correlation coefficient and

‡ *P*-value for two-tailed Student's *t* test of Individual dataset (Oncomine database).

### Mechanism of E1A-mediated downregulation of AXL

**Figure 4:**
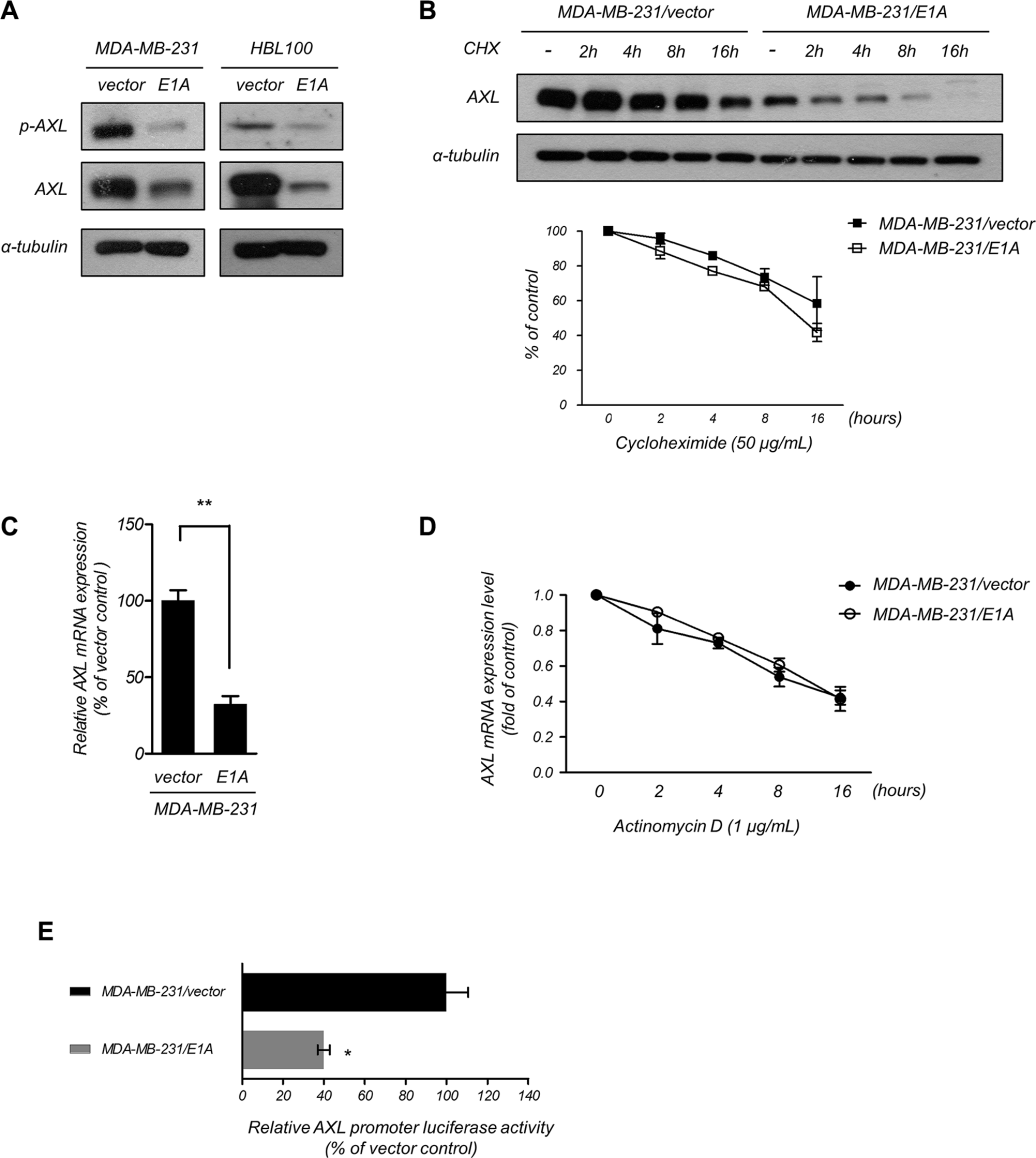
E1A transcriptionally downregulates AXL (**A**) Phosphorylation of AXL and AXL expression was assessed in E1A-expressing and vector-expressing MDA-MB-231 and HBL100 cells using western blotting analysis. α-Tubulin was used as an internal control. (**B**) The protein level of AXL was examined using western blotting analysis in MDA-MB-231/vector and MDA-MB-231/E1A cells treated with cycloheximide (50 μg/ml, CHX) at the indicated times and quantified (bottom). α-Tubulin was used as the internal protein loading control. The results are representative of at least 3 independent experiments. Bars indicate means ± s.e.m. (**C**) The relative *AXL* mRNA expression of MDA-MB-231/vector and MDA-MB-231/E1A cells was analyzed using qRT-PCR. The data were normalized to *GAPDH* mRNA levels in each sample. The columns represent the mean values from 3 independent experiments. Bars indicate the means ± s.e.m. **P < 0.01. (**D**) The mRNA level of AXL was determined using qRT-PCR in MDA-MB-231/vector and MDA-MB-231/E1A cells treatment with actinomycin D (1 μg/ml) at the indicated time points, and quantities were presented as fold changes compared with MDA-MB-231/vector cells. (**E**) Luciferase reporter assays used −1,251 to +475 flanking the TSS of *AXL* gene. The MDA-MB-231/vector and MDA-MB-231/E1A cells were transfected with the promoters, and luciferase activity was measured after transfection for 48 h. The pTK-*Renilla* plasmid was used as an internal control. Bars indicate the means ± s.e.m. *P < 0.05.

AXL has been reported to be involved in resistance to EGFR-TKI and was correlated with poor prognosis [26, 33], and E1A has been shown to downregulate the expression of AXL [34]. To further explore whether AXL was involved in E1A-mediated EGFR-TKI sensitization of breast cancer cells, we examined the phosphorylation and expression of AXL in E1A-transfected breast cancer cells. As shown in Figure 4A, ectopic expression of E1A in MDA-MB-231 and HBL100 cells significantly decreased the expression of AXL compared with the vector control cells. We next study the mechanism involved in E1A-mediated AXL inhibition. Using treatment of cycloheximide to inhibit *de novo* protein synthesis, we examined the protein stability of AXL with overexpression of E1A and found that the stability of AXL protein had no significant difference between MDA-MB-231/vector and MDA-MB-231/E1A cells (Figure 4B). Because the *AXL* mRNA level was lower in MDA-MB-231/E1A cells compared with MDA-MB-231/vector cells (Figure 4C). MDA-MB-231/vector and MDA-MB-231/E1A cells were treated with actinomycin D to block *de novo* RNA synthesis, and *AXL* mRNA expression was then determined by quantitative RT-PCR (qRT-PCR) analysis. We found that the stability of *AXL* mRNA was not significantly changed in MDA-MB-231/vector and MDA-MB-231/E1A cells (Figure 4D). To further study the transcriptional regulation involved in inhibition of AXL, we generated a 1726 bp *AXL* promoter construct containing nucleotides −1,251 to +475 (relative to the transcription start site as +1 of NM_021913) of the AXL gene and performed luciferase reporter assays. As shown in Figure 4E, expression of E1A significantly suppressed luciferase reporter activity in cells transfected with the AXL reporter. Moreover, to identify potential transcription factor involved in AXL regulation, we predicted the potential transcription factor binding to AXL promoter by TESS 2.0/TFSEARCH software (Supplementary Table S2 and Supplementary Figure S2A). Among the predicted candidates, we found seven transcription factors that have been reported to be regulated by E1A regulation [35–40] including FOS, JUN, ATF-1, CREB, YY1, C/EBPα, and Sp1 (Supplementary Table S2 and Supplementary Figure S2A). We used shRNA to block the E1A-induced expression of these transcription for further investigation on AXL promoter activity (Supplementary Figure S2B–S2H). However, only the knockdown of C/EBPα or Sp1 slightly enhanced AXL promoter activity in MDA-MB-231/E1A cells, whereas knocking down other transcription factors had no significant effects (Supplementary Figure S2I). These results suggest that the C/EBPα and Sp1 are partially involved in E1A-mediated transcriptional repression of AXL. However, we do not exclude that additional transcription factors may also contribute to AXL regulation. These results suggest that E1A downregulates AXL expression through transcriptional regulation.

### Downregulation of AXL is required for E1A-mediated suppression

**Figure 5:**
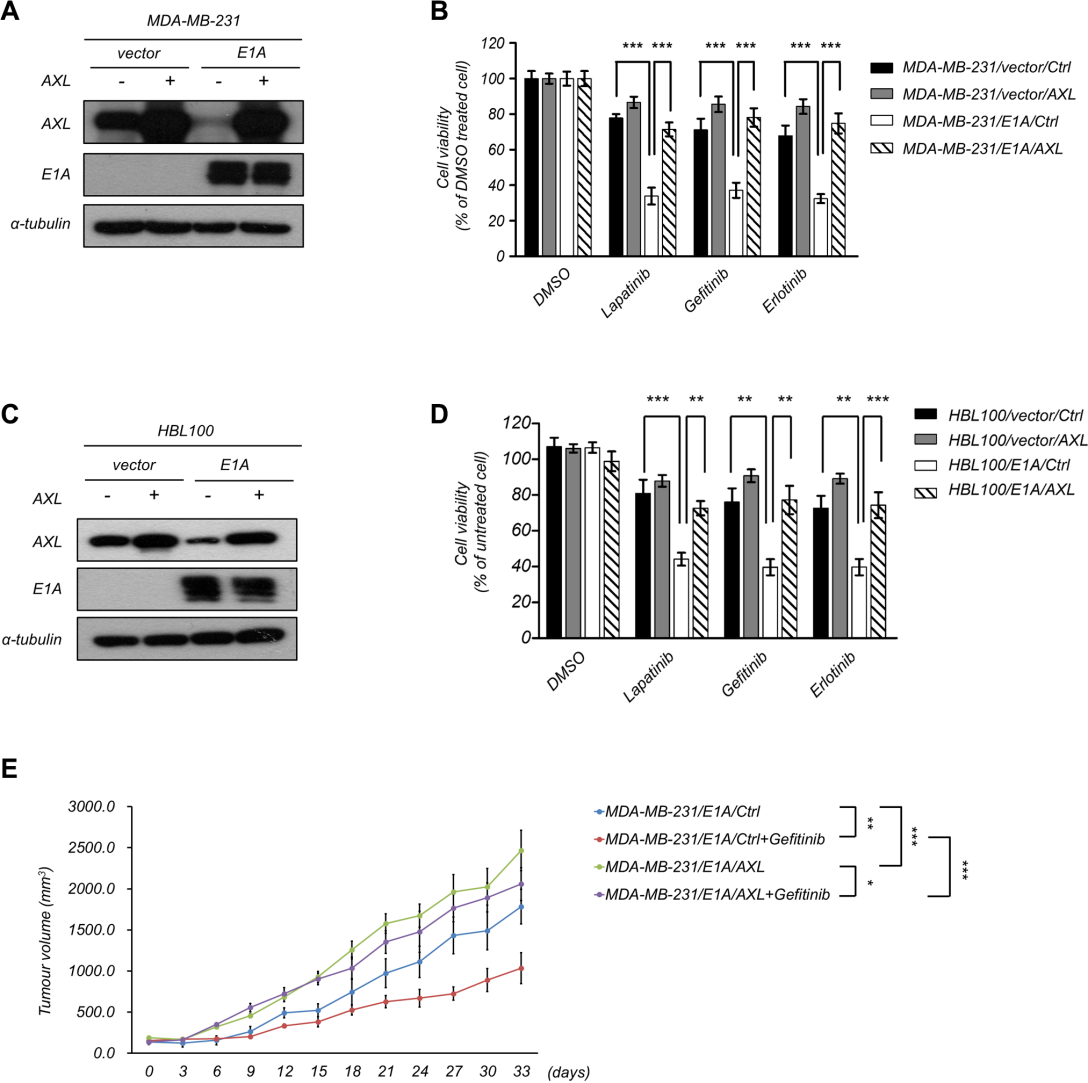
Role of AXL in E1A-mediated tumor suppression (**A–B**) Overexpression of AXL or a control vector in E1A-expressing cells (MDA-MB-231/E1A/AXL) and vector control cells (MDA-MB-231/vector/AXL). Western blotting analysis and MTT assays were performed to detect AXL and E1A expression and cell proliferation. α-Tubulin was used as an internal control. (**C**) Overexpression of AXL or control vector in HBL100/vector cells and HBL100/E1A cells. Western blot analysis was performed to detect AXL and E1A expression. α-Tubulin was used as internal control. (**D**) The cell viability was analyzed by MTT assay after treatment with EGFR-TKIs of 2.5 μM lapatinib, 10 μM gefitinib, and 20 μM erlotinib for 48 h. The columns are the mean values from 3 independent experiments. Bars indicate means ± s.e.m. ***P < 0.001. (**E**) MDA-MB-231/E1A/control or MDA-MB-231/E1A/AXL cells were orthotopically injected into SCID mice. Tumor volumes measured after combination treatment with gefitinib every three days are showed (*n* = 6 per group). Results are represented as the means ± s.e.m. *P < 0.05, **P < 0.01, ***P < 0.001.

To examine the functional role of AXL in E1A-mediated EGFR-TKI sensitization, we restored the expression of AXL in MDA-MB-231/E1A and HBL100/E1A cells and determined the effects on EGFR-TKI-induced cell death. Overexpression of AXL in MDA-MB-231/E1A and HBL100/E1A cells significantly abolished E1A-induced EGFR-TKI sensitization (Figure 5A–5D), indicating that expression of AXL is important for E1A-mediated EGFR-TKI sensitization. To further determine whether AXL is involved in E1A-mediated EGFR-TKI sensitization *in vivo*, we performed an orthotopic breast tumor growth assay in an animal model. MDA-MB-231/E1A/Ctrl and MDA-MB-231/E1A/AXL cells were orthotopically injected into the mammary fat pads of SCID mice, and tumor growth was measured. Four weeks after implantation of the cells, mice were treated with gefitinib (100 mg/kg/day) by oral gavage, and the tumor volumes were measured. After gefitinib treatment, the tumor volumes of MDA-MB-231/E1A/AXL-injected mice were higher than those of MDA-MB-231/E1A/Ctrl-injected mice, suggesting that downregulation of AXL is essential for E1A-mediated sensitization of EGFR-TKI (Figure 5E). Taken together, these results indicate that E1A reduces AXL expression and consequently results in enhanced EGFR-TKI sensitization of breast cancer cells both *in vitro* and *in vivo*.

## DISCUSSION

In the past few years, E1A has been shown to be a tumor suppressor that enhances the anti-neoplastic effect of several chemotherapeutic drugs through regulation of apoptotic molecules. Although a clinical trial of E1A gene therapy using tgDCC-E1A (E1A-lipid complex) combined with paclitaxel treatment for patients with platinum-resistant ovarian cancer was terminated at phase I/II of the study due to low accrual (ClinicalTrials.gov Identifier: NCT00102622), the results will be valuable for developing an efficient combination of E1A gene therapy with other agents in the future. Moreover, the toxicity was tolerable and the maximum tolerated dose (MTD) was determined. However, given the value of its application in cancer treatment, more studies and examination of its therapeutic effects should be carried out in the future.

Inhibition of EGFR was considered a potential therapeutic strategy for breast cancer patients because EGFR is commonly overexpressed in approximately 30% of breast cancers [2]. Unfortunately, several clinical tests demonstrated a poor response of breast cancer patients to EGFR-TKI. In contrast to lung adenocarcinomas, which often have EGFR kinase domain mutations that confer resistance to EGFR-TKI treatment, EGFR mutations are lacking or diverse in different clinical breast cancer datasets [4]. In addition, the EGFR-TKI resistance mechanisms of multiple cancers, including breast cancer, may be due to compensatory activation of other receptor tyrosine kinases, such as MET, AXL or IGF1R, which are overexpressed and activate downstream survival pathways (such as AKT, NF-κB and ERK) to help cancer cells escape from cell death following erlotinib or gefitinib treatment, as observed in lung cancer cells [10, 32, 41–43]. AXL also transactivates and phosphorylates EGFR and subsequently promotes cell proliferation and resistance to EGFR-TKI [11]. Furthermore, it has been shown that crosstalk between diverse kinases may cause EGFR-TKI resistance, especially ligand-independent AXL activation in breast cancer cells [30]. Therefore, to increase the efficiency of the clinical treatment, it is necessary to further explore the mechanism of EGFR-TKI response of breast cancer and assess multi-kinase inhibitors for combinational therapy, such as E1A gene therapy. Our results demonstrate that E1A gene therapy can sensitize breast cancer cells to EGFR-TKI both *in vitro* and *in vivo*, suggested that E1A may improve the current poor prognosis in breast cancer patients, and it may be a useful strategy to increase the anti-tumor activity of EGFR-TKI in breast cancer patients.

In addition to EGFR-TKI, AXL also induced resistance to other targeted therapies or chemotherapy in multiple types of cancer. Overexpression of AXL promotes cisplatin resistance through regulation of c-ABL expression in esophageal cancer [28]. Downregulation of AXL through the IL6/STAT3 pathway resulted in overcoming taxol resistance in ovarian cancer cells [44]. Moreover, high levels of AXL are involved in crizotinib (ALK inhibitor) resistance in lung cancer patients [45] and contribute to FLT3 inhibitor resistance in acute myeloid leukemia (AML) [46]. These studies suggest that AXL plays an important role in drug resistance.

## MATERIALS AND METHODS

### Cell lines and DNA constructs

Human breast cancer cell lines (MDA-MB-231, HS578t, HBL100, MCF7, T47D, MDA-MB-453, and MDA-MB-468) were purchased from the American Type Culture Collection (ATCC). All cells were testing for mycoplasma contamination and were authenticated by STR profiling at the Bioresource Collection and Research Center (Hsinchu, Taiwan) and the Center for Genomic Medicine, NCKU (Tainan, Taiwan) using an AmpFℓSTR^®^ Identifier^®^ PCR Amplification Kit (Applied Biosystems Cat.# 4322288), which analyzed 16 STR loci. All cells were frozen within 1–2 weeks after authentication. MDA-MB-231, HS578t, and MDA-MB-453 cells were cultured in Dulbecco's modified Eagle's medium (DMEM)/F12. MCF-7 and T47D cells were grown in RPMI-1640 medium. MDA-MB-468 and HBL100 cells are grown in DMEM. Mediums were supplemented with 10% FBS, 100 units/mL penicillin and 100 μg/ml streptomycin. MDA-MB-231 and its E1A/vector-stable transformants have been described previously [47] and were grown under the same conditions as the control with G418 supplements. The lentiviral AXL shRNA clones TRCN0000001040 and TRCN0000195353, Full-length human AXL (NM_021913) was amplified by PCR, and the primer sequences are shown in Supplementary Table S1.

### Reagents and antibodies

Gefitinib, erlotinib, lapatinib and R428 were purchased from Selleck Chemicals (Houston, TX, USA). Propidium iodide and RNase A were purchased from Sigma-Aldrich (St Louis, MO, USA). The primary antibodies used were as follows: E1A (ab-52523; Abcam), EGFR (sc-03; Santa Cruz Technology), AXL (#4566; Cell Signaling Technology), p-AXL (#5724; Cell Signaling Technology), and α-Tubulin (T-5168; Sigma). All secondary antibodies were purchased from Jackson ImmunoResearch Laboratories, Inc. (West Grove, PA, USA).

### Cell viability by MTT assay

The cells were seeded in 96-well plates at a density of 1 × 10^4^ cells per well overnight and then treated with the indicated drug concentrations. Each treatment was tested in triplicate wells. After incubation for 48 h, the MTT reagent was added at a final concentration of 1 μg/μl to each well and incubated for 3 h. Finally, DMSO was added to dissolve the purple crystals, and the absorbance of the plate was measured at 570 nm by an ELISA plate reader.

### Cell apoptosis by flow cytometry analysis

Aliquots of 3 × 10^5^ cells were harvested and washed with PBS and then fixed in 70% ethanol on ice overnight. After fixation, cells were washed twice with PBS and stained in PBS containing 1 μg/ml of propidium iodide (Sigma) and RNase for 1 h. Samples were then analyzed by flow cytometry with the FACSCalibur flow cytometer (BD Biosciences).

### Cell apoptosis by Annexin V-FITC/PI double staining assay

Cells were seeded in 6-well plates at a density of 3 × 10^5^ cells per well overnight and then treated with the indicated drug concentrations. Cells were harvested and washed twice with PBS and resuspended in Annexin V binding buffer. Annexin V FITC (BioLegend) and propidium iodide (Sigma) double stained methods were performed according to the manufacturer's instructions and analyzed by flow cytometry with the FACSCalibur flow cytometer (BD Biosciences).

### Orthotopic breast tumor growth assay

Four-week-old female severe combined immunodeficient (SCID) mice were supplied by the National Laboratory Animal Center (Taipei, Taiwan) and were used for the orthotopic xenograft studies. According to the 3Rs (replacement, reduction and refinement), there were six mice per group. For the tumor growth assay, 5 × 10^6^ tumor cells were mixed with Matrigel (BD Biosciences, San Jose, CA, USA) and injected into the fat pads. When the tumor volumes reached approximately 100 mm^3^, as determined by measuring tumor length and width using calipers and calculating the volume by the formula (1/2 [length **×** width^2^]), mice were randomly allocated into eight groups to receive gefitinib (100 mg/kg/day) or vehicle by oral gavage [48], and tumor volumes were measured every three days. All animal work was performed using a protocol approved by the National Health Research Institutional Animal Care and Use Committee.

### RNA isolation, RT-PCR and qRT-PCR assay

Total RNA was extracted from cells with TRIzol reagent (Roche Diagnostics, Mannheim, Germany) and reverse transcribed into cDNA with MMLV reverse transcriptase (Invitrogen). The reactions were incubated at 37°C for 50 min, 72°C for 10 min, and 4°C for 5 min. qRT- PCR was performed using a Roche LightCycler 480 qRT-PCR system. All reactions were carried out in triplicate.

### Promoter constructs and luciferase reporter assays

The 1,726 bp *AXL* promoter (nucleotides −1,251 to +475 were relative to the transcription start site as +1 of NM_021913) was amplified by PCR. In the PCR protocol, samples were first denatured at 94°C for 5 min, followed by 30 cycles of 94°C for 30 s, 55°C for 1 min and 72°C for 3 min, with a final extension at 72°C for 10 min. The primer pairs for constructs (p1627-p480) of the *AXL* promoter are shown in Supplementary Table S1. The sequence of these constructs was confirmed by DNA sequencing. Luciferase activities were analyzed with the Luciferase Assay System (Promega) as described previously [49]. The firefly luciferase reporter gene construct and the pTK-*Renilla* luciferase construct (for normalization) were cotransfected (1 μg each) into each well. After transfection for 48 h, luciferase activity was measured using the Dual-Luciferase Reporter Assay System (Promega).

### Breast tumor specimens

The breast cancer tissues were from the Tissue Bank Core Facility for Genomic Medicine of the National Taiwan University Hospital (Taipei, Taiwan) and were obtained with Institutional Review Board approval. Patients had not received neoadjuvant chemotherapy or radiation therapy. Patients who died within 30 days of surgery were excluded from the survival analysis. Survival was calculated from patients whose survival information was available for analysis (*n* = 73).

### Statistical analysis

All data were analyzed by GraphPad Prism 6 software (La Jolla, CA, USA). Data were approximately normally distributed and are presented as the mean ± s.e.m. from at least three independent experiments. Statistical analysis among the experimental groups was conducted using a two-tailed Student's *t* test or one-way ANOVA. Survival curves were generated using the Kaplan-Meier method, and the log-rank test was used to test the difference in survival curves. A *P*-value of less than 0.05 was considered statistically significant.

## SUPPLEMENTARY MATERIALS TABLES AND FIGURES


