# Supplementary material for: A novel application of E1A in combination therapy with EGFR-TKI treatment in breast cancer
Source: Oncotarget. 2016 Aug 31;7(39):63924–36. doi: 10.18632/oncotarget.11737 (PMC5325414; doi:10.18632/oncotarget.11737)
Supplement: Supplementary file 1 [file oncotarget-07-63924-s001.pdf]

A novel application of E1A in combination therapy with EGFR-TKI treatment in breast cancer

Supplementary Materials

Supplementary Table S1: Sequences and information of PCR primers for cloning

**Cloning primer**
AXL
5′-CCCAAGCTTGGAAAGTTTGGCACCCATG-3′ (forward)
5′-CCCAAGCTTGGTTGTCTCAGGCACCATC-3′ (reverse)
**AXL p1726 reporter**
5′-gggctagcGACACAGCCCAGGGAGACAACG-3′ (forward)
5′-ggctcgagGGGTGCCAAACTTTCCTCAGAA-3′ (reverse)
**qRT-PCR primer**
AXL
5′-CGTAACCTCCACCTGGTCTC-3′ (forward)
5′-TCCCATCGTCTGACAGCA-3′ (reverse)

Supplementary Table S2: List of Transcription factors used in Supplementary Figure S2

E1A-regulated TFs (Reference-based) (*n* = 34)	Predicted TFs binding to AXL promoter (*n* = 33)
AP-1	AP-1
ATF-1	AP-2
C/EBPα	AP-2alpha
C/EBPβ	ATF
CREB	C/EBPalpha
CtBP	c-Myc
DREF	CPEbind
DRTF1	CREB
DYRK	CRE-BP1
E2F1	Da
E2F2	E2
E2F3	Egr-1
E4F	ER
ETF	GATA-1
FOXK1/K2	GBF2
Foxo3a	GCN4
hBre1	GLO
MEF2	GR
P/CAF	HOXA4
p21	Krox-20
p300	MEB-1
p400	MIG1
p50	NF-1
p53	Oct-1
p65	RAP1
RB	RAR-alpha
RFX1 (EF-C)	REVErbA
Sp1	Sp1
TBP	T3R
TFIIB	TEC1
TFIID	Tra-1
TFIIIC	WT1
TRRAP	YY1
YY1	

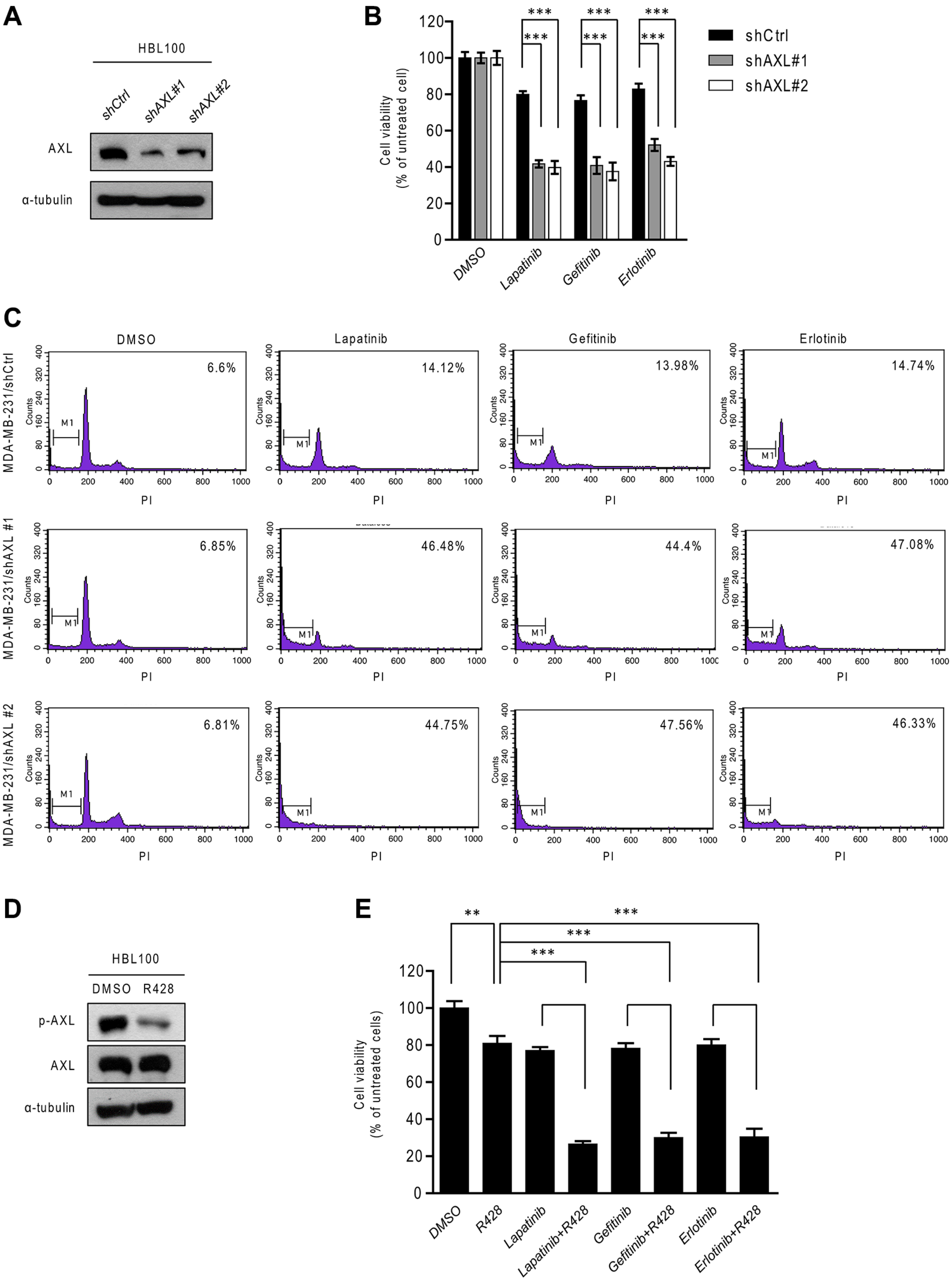

Supplementary Figure S1: AXL is critical for the sensitization of EGFR-TKIs. (**A**) Knockdown of AXL in HBL100 cells was confirmed by using western blotting analysis. α-tubulin was used as the internal protein loading control. (**B**) The cell viability was analyzed using MTT assay after treatment EGFR-TKIs. The columns are the mean values from 3 independent experiments. Bars indicate the means ± s.e.m. ***P* < 0.01. (**C**) Representative DNA histograms of PI staining of Figure 2C (**D**–**E**) Effects of the treatment of AXL inhibitor, R428 (10 nM) for 48 h on EGFR-TKI sensitivity. (D) Protein expression of both phosphorylated and total AXL were analyzed using western blotting analysis. (E) The cell viability were analyzed by MTT assay after treatment with EGFR-TKIs. The columns are the mean values from 3 independent experiments. Bars indicate means ± s.e.m. **P* < 0.05, ***P* < 0.01, ****P* < 0.001.

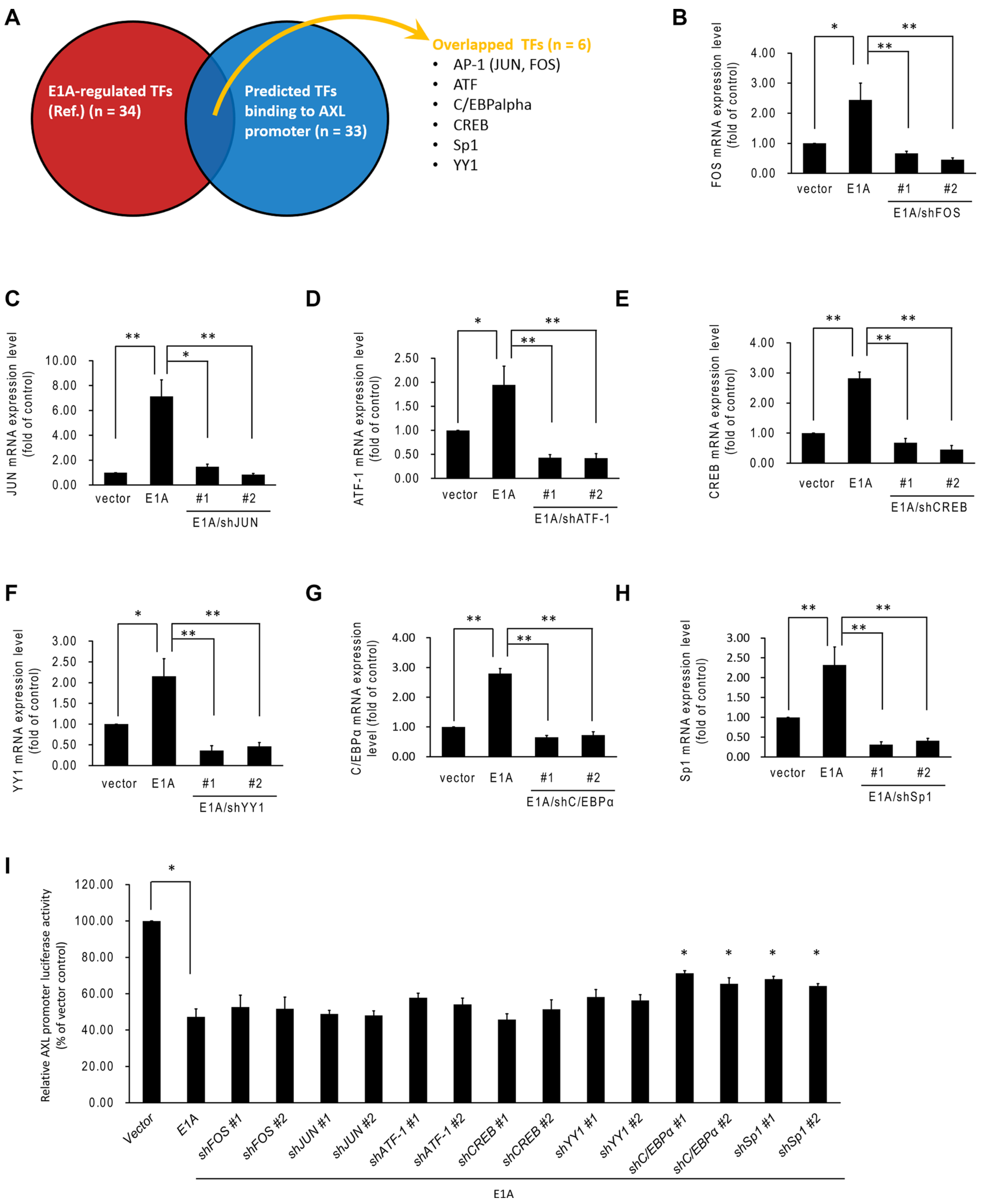

Supplementary Figure S2: Combination of E1A-regulated transcription factors and predicted transcription factors binding to AXL. (**A**) E1A-regulated transcription factors (TFs) was showed in left red circle. AXL promoter sequence was analyzed by TESS 2.0 and thirty three candidates were showed in right green circle. Only six overlapped TFs were identified. (**B–I**) Roles of FOS, JUN, ATF-1, CREB, YY1, C/EBPα and Sp1 in regulating Axl expression. (B–H) Knockdown of E1A-induced FOS, JUN, ATF-1, CREB, YY1, C/EBPα and Sp1 expression in MDA-MB-231/E1A cells were confirmed by qRT-PCR assay. The AXL promoter luciferase activity were then assayed (I). The columns are the mean values from 3 independent experiments. The pTK-Renilla plasmid was used as an internal control. Bars indicate means ± s.e.m. $*P < 0.05$, $**P < 0.01$
